# Clinical Efficacy of Epidural Injections of Local Anesthetic Alone or Combined with Steroid for Neck Pain: A Systematic Review and Meta-Analysis

**DOI:** 10.1155/2022/8952220

**Published:** 2022-05-26

**Authors:** Bang-zhi Li, Wen-hai Tang, Yang Li, Lei Zhou, Ming-guo Liu, Sheng-Xue Bao

**Affiliations:** ^1^College of Acupuncture and Orthopedics, Hubei University of Chinese Medicine, Wuhan 430061, China; ^2^The Affiliated Hospital of Hubei University of Traditional Chinese Medicine, Wuhan 430070, China; ^3^Department of Spine Surgery, Second Affiliated Hospital of Hainan Medical College, Haikou 570100, China; ^4^Department of Anesthesiology, Hubei Provincial Hospital of Traditional Chinese Medicine, Wuhan 430061, China; ^5^Hubei Provincial Academy of Traditional Chinese Medicine, Wuhan 430070, China; ^6^Department of Anesthesiology, Hubei Provincial Armed Police Corps Hospital, Wuhan 430060, China

## Abstract

**Aims:**

To compare the effectiveness of cervical epidural injections of local anesthetic with vs. without a steroid.

**Methods:**

Three databases (PubMed, Embase, and Cochrane library) were used to search and assess all clinical randomized controlled trials regarding the clinical efficacy of epidural injections from January 01, 2009, to October 31, 2020. Cochrane review criteria and the Interventional Pain Management Techniques-Quality Appraisal of Reliability and Risk of Bias Assessment instrument were used to evaluate the methodologic quality of the included studies. Qualitative and quantitative analyses were performed according to best evidence synthesis principles and by single-arm meta-analysis, respectively.

**Results:**

Based on the search criteria, 4 RCTs were qualitatively and quantitatively analyzed in the single-arm meta-analysis. Treatment with lidocaine alone or with the steroid resulted in decreases of 4.46 and 4.29 points, respectively, in pain scores and of 15.8 and 14.46 points, respectively, in functional scores at 6 months. Similar trends were observed at the 1-year follow-up: pain scores decreased by 4.27 and 4.14 points, while functional scores decreased by 15.94 and 14.44 points in patients with neck pain who received lidocaine without or with the steroid, respectively. In the 3 studies that reported 2-year follow-up data, patients with neck pain treated with lidocaine or lidocaine + steroid showed 4.2- and 4.14-point decreases, in pain score and 15.92- and 14.89-point decreases, respectively, in functional scores.

**Conclusions:**

The studies showed level I (strong) evidence for short- and long-term improvements in pain relief and functionality with cervical epidural injections of local anesthetic alone or with a steroid in the management of neck pain.

## 1. Introduction

Degenerative cervical spine lesions and cervical postoperative syndrome are the leading causes of neck pain, including cervical disc herniation and cervical stenosis, which bring higher economic burdens and disability rates to society [1–5]. Chronic cervical pain not only increases the burden of life on patients but also increases the psychological burden of patients and now ranks third among conditions that contribute to disability [6]. Current clinical treatment options for neck pain include conservative and surgical treatment. Conservative treatment is mainly oral drugs and physical therapy. However, conservative treatment is generally ineffective in the treatment of refractory neck pain, oral medication will increase the burden on the gastrointestinal tract, and adverse reactions such as gastric ulcers and bleeding may occur. However, the rate of reoperation due to the failure of surgical interventions is 32% [7–17]. Cervical epidural injections have been widely used to manage neck pain [18], especially in patients who are poor candidates for surgery [19, 20]. Although a number of randomized trials have investigated the efficacy of cervical epidural injections of local anesthetics alone or in conjunction with steroids, the long-term effectiveness of these treatments in managing chronic neck pain is controversial [8, 10, 12, 21–28].

Steroids are used in cervical epidural injections to control inflammation and suppress edema of the nerve root. In a preliminary report of a systematic review and meta-analysis of the efficacy of fluoroscopically guided cervical epidural steroid injection for the treatment of radicular pain, improvements in vital functions were reported in 58% of patients at 2 months [29]. The mechanisms of action of steroids include suppression of ectopic discharges from inflamed nerves as well as proinflammatory cytokines, improvement of blood flow, and lysing of iatrogenic and inflammatory adhesions [30]. Besides neck pain, steroids are widely used to manage painful diseases including osteoarthritis and gout and are typically combined with local anesthetics to achieve greater efficacy. However, local anesthetics alone can have a comparable effect in terms of pain relief, and there is no evidence that this is enhanced by the addition of steroids. Some studies have reported similar degrees of pain relief and functional improvement in patients with neck pain secondary to disc herniation or postsurgery syndrome who were treated by cervical epidural injections of local anesthetics without or with steroids [31–35].

In order to address this controversy, we carried out a systematic review and meta-analysis evaluating the long-term efficacy of cervical epidural injections with a local anesthetic alone or combined with a steroid in the management of neck pain.

## 2. Methods

### 2.1. Study Identification and Search Strategy

The PubMed (http://www.ncbi.nlm, http://nih.gov/pubmed), Embase (http://www.embase.com), and Cochrane library (http://www.thecochranelibrary.com) databases were searched for studies published between January 2009 to October 2020. The following search terms were used: (((((“injections, epidural” OR ((((((((((Extradural Injections OR Peridural Injections OR AND Peridural OR ((“injections” OR “injections” OR “injection” [All Fields]) AND (“Neck Pain” OR ((((((((((((((((((((((((((((((((((((((((Neck Pains OR Pain, Neck OR ((“pain” OR “pain” OR “pains”) AND Neck)) OR Neck Ache AND Cervical OR Posterior Cervical Pain AND Anterior Cervical) OR Anterior Neck Pain OR (Anterior [All Fields] AND Neck Pains)) OR ((“neck pain” [MeSH Terms] OR (“neck” AND “pain”) OR “neck pain”) AND Anterior) OR ((“neck pain” OR (“neck” [All Fields] AND “pain”) OR “neck pain” OR (“neck” AND “pains”) OR “neck pains”) AND (“2009/01/01” [PDAT]: “2020/10/20” [PDAT])))) AND (((((randomized controlled trial [Title/Abstract]) OR randomized [Title/Abstract]) OR placebo [Title/Abstract])) OR (((((((((Health Care Category [Title/Abstract]) OR (Environment [Title/Abstract] AND Public Health [Title/Abstract])) OR Public Health [Title/Abstract]) OR Epidemiologic Methods [Title/Abstract]) OR Epidemiologic Study Characteristics [Title/Abstract]) OR Epidemiologic Studies [Title/Abstract]) OR Case-Control Studies [Title/Abstract])) OR “Retrospective Studies”[MeSH])).

### 2.2. Study Selection

All studies that described the management of chronic neck pain and included outcome evaluations over a period of at least 6 months were reviewed. All randomized trials in all languages with appropriate statistical analyses were included. Study type: randomized controlled trial (RCT). Patients: all patients with neck pain secondary to cervical disc herniation, spondylosis, cervical, or postsurgery syndrome treated with cervical epidural injections of local anesthetic alone or in conjunction with a steroid. Intervention: cervical interlaminar injections of anesthetic (lidocaine) and steroid (betamethasone). Outcome: for pain relief, a 50% decrease from the baseline pain score or a change of at least 3 points on an 11-point pain scale was considered clinically significant. For functional status improvement, a change of ≥30% in disability score or 50% improvement from baseline was considered clinically significant. A study was judged to be positive if the effectiveness of the therapy was demonstrated through comparison with a control group or from baseline to follow-up. A negative study was defined as one in which no difference was seen as a result of the treatment or in which there was no measurable improvement from baseline. Reference point measurements were at 6 months, 1 year, and 2 years. Book chapters, case reports, and reports without a definitive diagnosis were excluded. Studies in which patients had acute trauma, fractures, malignancies, and inflammatory diseases were also excluded.

### 2.3. Data Collection

Two investigators independently performed the initial search and completed study screening and data extraction according to the selection criteria. Disagreements were resolved through discussion between 2 investigators; a third investigator was consulted in cases where a consensus could not be reached. Data synthesis and analysis, including assessment of study quality, were performed by the 2 investigators, with a third investigator consulted as needed.

### 2.4. Methodological Quality of Studies

Cochrane review and the Interventional Pain Management Techniques Quality Appraisal of Reliability and Risk of Bias Assessment (IPM-QRB) criteria were used to evaluate the quality of each individual article for RCTs. Studies meeting at least 9 of the 13 Cochrane review inclusion criteria were considered to be of high quality; those meeting 5–8 criteria were deemed to be of moderate quality; and those meeting <5 criteria were low-quality studies that were excluded. Studies meeting the IPM-QRB inclusion criteria with a score of 32–48 were considered to be of high quality and were included in the analysis; those with a score of 16–31 were judged as being of moderate quality; and those meeting the inclusion criteria but with a score < 16 were low-quality studies that were excluded.

The methodologic quality and internal validity of each publication, as well as the quality of evidence, were independently assessed in an unblinded, standardized manner by 2 investigators. In the case of any disagreements, a third investigator performed the assessment and a consensus was reached. Outstanding issues were resolved through a discussion involving all investigators. The evidence was analyzed based on best-evidence synthesis principles and was modified and collated according to multiple criteria including Cochrane review and United States Preventive Services Task Force criteria (Table 1). The analysis was conducted based on 5 levels of evidence ranging from strong to opinion- or consensus-based. The results of best evidence as determined by the evidence level were used. If there were any conflicts of interest (e.g., authorship), the investigator in question was recused from the review of evidence.

### 2.5. Statistical Analysis

The single-arm meta-analysis was performed using Comprehensive Meta-analysis v3.0 (Biostat, Englewood, NJ, USA). The *I*^2^ statistic was used to assess the heterogeneity of included studies. Data were displayed as forest plots to evaluate treatment effects. Pain and functional status improvement data from the included studies are reported as standardized mean differences with 95% confidence interval. All analyses were based on treatment modality and the injected solution. Short- and long-term improvement was defined as any improvement at 6 months and after 6 months, respectively.

## 3. Results

### 3.1. Study Selection

**Figure 1:**
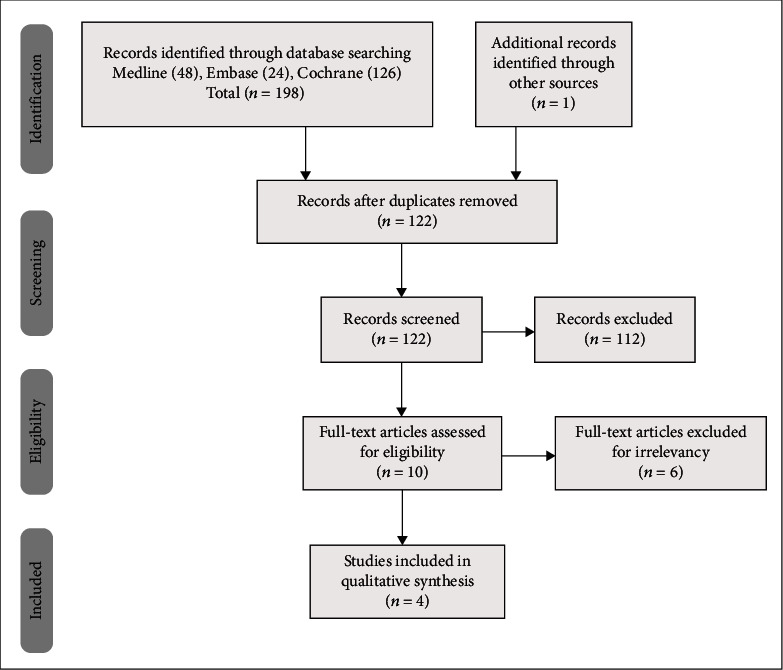
Flow diagram illustrating published literature evaluating epidural injection in neck pain.

A flow diagram of the study selection process according to PRISMA guidelines is shown in Figure 1. Based on the search criteria, 10 publications were considered for inclusion; 6 of these were excluded because of duplicate publications or lack of data. Ultimately, 4 RCTs [31, 32, 34, 36] were included in the present analysis.

### 3.2. Methodologic Quality and Risk of Bias Assessment

The results of the methodologic quality and risk of bias assessments for each of the included studies are shown in Tables 2 and 3. According to Cochrane review and IPM-QRB criteria [37, 38], all of the RCTs were of high quality.

### 3.3. Study Characteristics

The characteristics and outcomes of the included studies are shown in Table 4. The studies were not heterogeneous. One RCT [36] followed up patients treated with epidural injections of a steroid (*n* = 30) or without the steroid (*n* = 30) for 1 year; 50% pain relief associated with a 50% functional improvement was considered significant. Work status was also an outcome measure; at the 1-year follow-up, the effectiveness in terms of pain relief and functional improvement was 71.5%. The second RCT [32] included 120 patients, and the interventions and outcome measures for each group were similar to those in the first RCT. The rates of effectiveness for pain relief and functional improvements were 72% and 68% in patients who received epidural injections without and with the steroid, respectively. The third and fourth RCTs had similar interventions and outcome measures as the first 2, but the follow-up time was 2 years. One study [34] showed improvements in pain and function after an average of 6 treatment sessions over a period of 2 years. In the other study [31], patients receiving epidural injections without the steroid experienced 65.6 ± 37.8 weeks of pain relief over a period of 2 years compared to 59.4 ± 34.2 weeks in those receiving injections that included the steroid, with no significant difference between groups.

### 3.4. Analysis of Study Quality

The quality of evidence of the included studies was assessed using a modified version of evidence grading [39] with high evidence (level I) from multiple relevant high-quality RCTs. All studies reported pain relief and functional improvement in patients who received epidural injections with or without the steroid. Conventional meta-analysis was not feasible because there were no significant differences between patients receiving epidural injections with lidocaine alone vs lidocaine + steroid. To assess pain relief and functional improvement, we performed a single-arm meta-analysis of the data from the 4 studies [31, 32, 34, 36].

### 3.5. Pain and Functionality at 6 Months, 1 Year, and 2 Years

**Figure 2:**
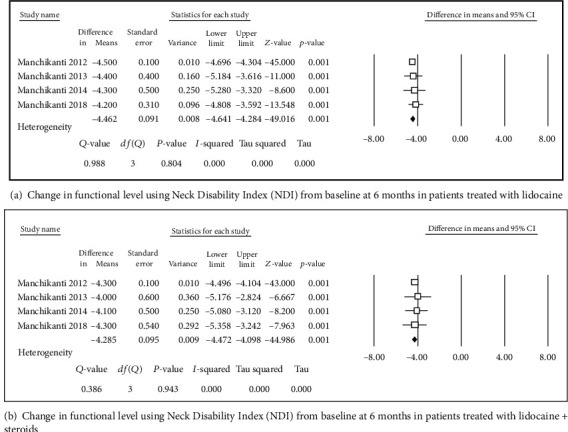
Change in functional level using Neck Disability Index (NDI).

**Figure 3:**
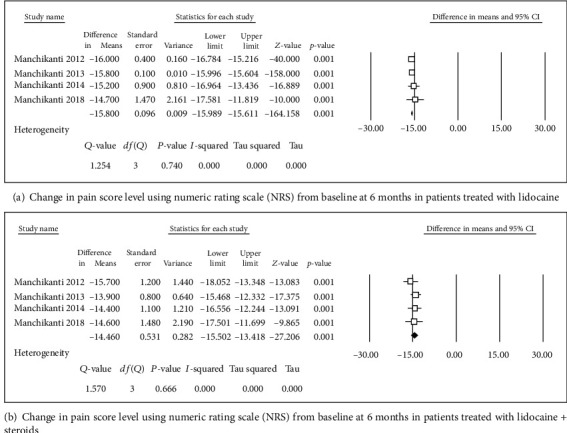
Change in pain score level using numeric rating scale (NRS).

**Figure 4:**
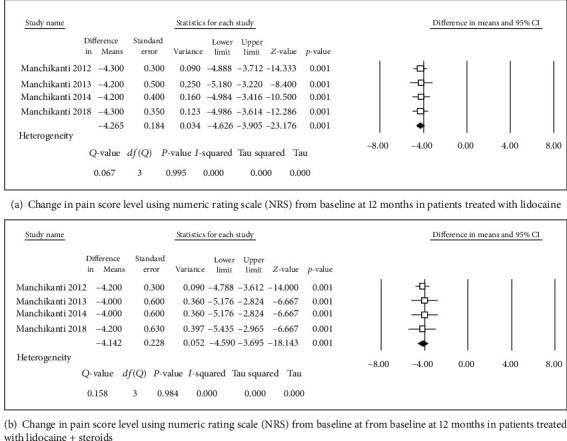
Change in pain score level using numeric rating scale (NRS).

**Figure 5:**
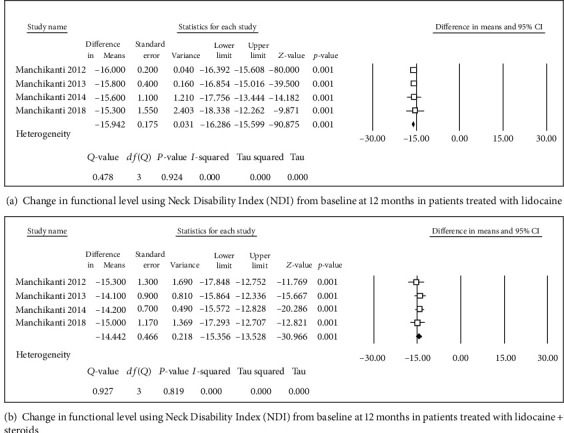
Change in functional level using Neck Disability Index (NDI).

**Figure 6:**
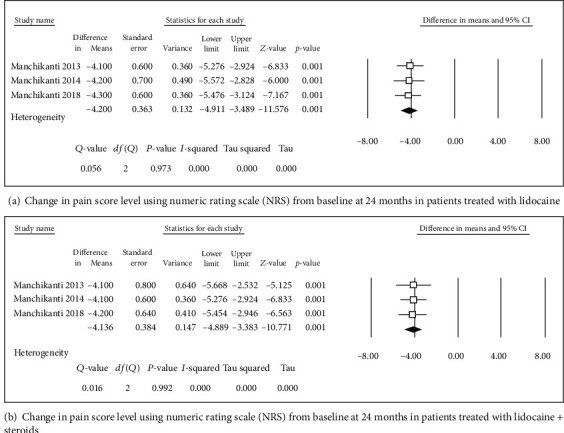
Change in pain score level using numeric rating scale (NRS).

**Figure 7:**
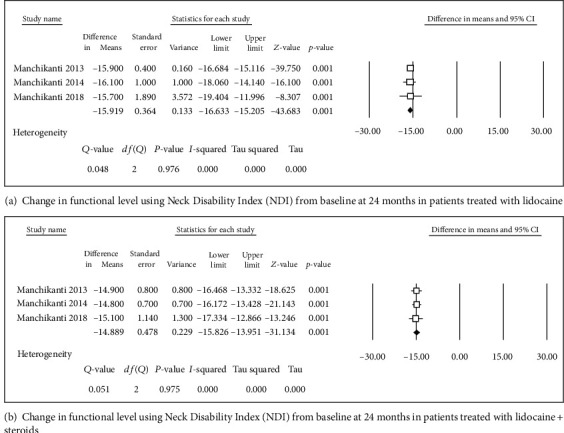
Change in functional level using Neck Disability Index (NDI).

Four studies [31, 32, 34, 36] were included in this single-arm meta-analysis of pain relief and functional improvement (Figures 2(a) and 2(b)). Treatment with lidocaine alone or with the steroid resulted in decreases of 4.46 and 4.29 points, respectively, in pain scores (Figures 3(a) and 3(b)) and of 15.8 and 14.46 points, respectively, in functional scores (Figures 2(a) and 2(b)) at 6 months. Similar trends were observed at the 1-year follow-up: pain scores decreased by 4.27 and 4.14 points (Figures 4(a) and 4(b)), while functional scores decreased by 15.94 and 14.44 points (Figures 5(a) and 5(b)) in patients with neck pain who received lidocaine without or with the steroid, respectively. In the 3 studies that reported 2-year follow-up data [31, 32, 34], patients with neck pain treated with lidocaine or lidocaine + steroid showed 4.2- and 4.14-point decreases, in pain score (Figures 6(a) and 6(b)) and 15.92- and 14.89-point decreases, respectively, in functional scores (Figures 7(a) and 7(b)).

## 4. Discussion

This systematic review and single-arm meta-analysis of 4 RCTs provided evidence that cervical epidural injections with lidocaine alone or in combination with the steroid betamethasone alleviated pain and improved functionality in patients with neck pain. According to Cochrane review and IPM-QRB criteria, all studies were of high quality. Furthermore, the studies included descriptions of sample size and employed similar injection approaches and pharmacologic agents, which increased the reliability and consistency, respectively, of reported outcomes. All of the studies demonstrated that both treatments were effective for the management of neck pain secondary to cervical disc herniation, spondylosis, or postsurgery syndrome. One of the studies [36] included 1-year follow-up data and the other 3 [31, 32, 34] followed up patients for 2 years, which reduced the bias in outcome reporting. Additionally, opioid intake by the patients was significant decreased by the 2 two treatments. However, there was no significant difference between the 2 treatments in terms of efficacy in any of the RCTs.

The cervical spine has two natural spaces in structure, namely, the cervical foramen and the cervical intervertebral space. Therefore, cervical injection treatment involves an epidural injection through the cervical intervertebral plate and an epidural injection through the cervical intervertebral foramen. The former is commonly used to treat central or paracentral or multisegment disc herniation, while the latter is primarily used for single-segment disc herniation [40–42]. Both have some complication rates, and many studies comparing interlamellar and transforaminal approaches to neck pain have shown a greater risk of neurological complications [19, 43–47], including infarction of the spinal cord, brainstem, brain, or cerebellum [40]. Potential complications of the interlamina approach include needle placement, infection, or the need for additional medication [2].

The most common causes of neck pain are cervical intervertebral herniation, spondylosis, or stenosis; facet joint, vertebral body, meningeal, blood vessel, nerve sheath, or nerve pathology; and postsurgery syndrome [32, 48–51]. Axial neck pain is associated with disc herniation, facet joint degeneration, cervical spondylosis, or ligamentous diseases. Given the relationship between axial neck pain and disc herniation with radiculitis and spinal stenosis, cervical epidural injections are used to manage axial neck pain [38]. Some patients have a long history of neck pain, which is difficult to manage from a clinical standpoint. Conservative treatments for chronic neck pain include oral analgesics or anti-inflammatory drugs or physical therapy, which can eliminate pain symptoms in some patients by up to 80% [9, 52–64]. Nevertheless, a subset of patients requires decompression surgery although this is not always an option because of the high cost and surgical contraindications. Besides surgery, cervical epidural injections are a valid treatment approach [10, 65, 66] that were shown to be effective in many studies [12, 19, 41, 43, 47].

Dexamethasone is a nonparticulate steroid while triamcinolone and betamethasone are particulate steroids [67]. The use of steroids is linked to the risk of spinal cord injury [67–70]. No significant differences in efficacy have been reported between the 2 types of steroid for the treatment of cervical radiculopathy [71]. Steroids can suppress ectopic discharges from inflamed nerves, improve blood flow, and induce the lysis of iatrogenic and inflammatory adhesions and proinflammatory cytokines.

A washout function has also been ascribed to local anesthetics [40, 72]. Thus, it is possible that the reason there were no differences observed between treatment with anesthetic alone or in conjunction with a steroid in the 4 RCTs is that both agents play the same roles in pain relief and functional improvement. In the evaluation of epidural anesthesia plus corticosteroids for the treatment of cervical arm radiculolargia [73], continuous epidural control of chronic cervical-arm pain was better compared with a single injection. Although both injections use corticosteroids. Thus, local anesthetics have an independent or additive effect.

## 5. Conclusions

This systematic review and single-arm meta-analysis of 4 RCTs showed strong (level I) evidence for the efficacy of fluoroscopic cervical epidural injections of a local anesthetic alone or combined with a steroid in the treatment of neck pain secondary to cervical disc herniation, spondylosis, stenosis, or postsurgery syndrome. Given the risks and adverse effects associated with both types of drug and potential interaction effects, additional studies are needed to determine whether the 2 treatments are equally effective, in which case the use of steroids can be avoided.

## Figures and Tables

**Table 1:** Qualitative modified approach to grading of evidence.

Level	Strength of evidence	Description
I	Strong	Evidence obtained from multiple relevant high-quality randomized controlled trials

II	Moderate	Evidence obtained from at least one relevant high-quality randomized controlled trial or multiple relevant moderate- or low-quality randomized controlled trials

III	Fair	Evidence obtained from at least one relevant moderate- or low-quality randomized controlled trial with multiple relevant observational studies OR Evidence obtained from at least one relevant high-quality nonrandomized trial or observational study with multiple moderate- or low-quality observational studies

IV	Limited	Evidence obtained from multiple moderate- or low-quality relevant observational studies

V	Consensus-based	Opinion or consensus of a large group of clinicians and/or scientists

**Table 2:** Methodologic quality assessment according to Cochrane review criteria.

	Manchikanti 2012 (36)	Manchikanti 2013 (37)	Manchikanti 2014 (38)	Manchikanti 2018 (39)
Adequate randomization	Y	Y	Y	Y
Concealed treatment allocation	Y	Y	Y	Y
Patient blinded	Y	Y	Y	Y
Care provider blinded	Y	Y	Y	Y
Outcome assessor blinded	N	N	N	N
Dropout rate described	Y	Y	Y	Y
All randomized participants analyzed in the group	Y	Y	Y	Y
Reports of the study free of suggestion of selective outcome reporting	Y	Y	Y	Y
Groups similar at baseline with respect to most important prognostic indicators	N	Y	N	N
Cointerventions avoided or similar	Y	Y	Y	Y
Compliance acceptable in all group	Y	Y	Y	Y
Time of outcome assessment in all groups similar	Y	Y	Y	Y
Other sources of potential bias unlikely	Y	Y	Y	Y
Score	11/13	12/13	11/13	11/13

Y: yes; N: no; U: unclear.

**Table 3:** Methodologic quality assessment using the Interventional Pain Management Techniques-Quality Appraisal of Reliability and Risk of Bias Assessment instrument.

	Manchikanti 2012 (36)	Manchikanti 2013 (37)	Manchikanti 2014 (38)	Manchikanti 2018 (39)
I. Trial design and guidance reporting				
1. Consort or spirit	3	3	3	3
II. Design factors				
2. Type and design of trial	2	2	2	2
3. Setting/physician	2	2	2	2
4. Imaging	3	3	3	3
5. Sample size	2	3	3	2
6. Statistical methodology	1	1	1	1
III. Patient factors				
7. Inclusiveness of population	2	2	2	2
8. Duration of pain	2	2	2	2
9. Previous treatments	2	2	2	2
10. Duration of follow-up with appropriate interventions	2	3	3	2
IV. Outcomes				
11. Outcome assessment criteria for significant improvement	4	4	4	4
12. Analysis of all randomized participants in the groups	2	2	2	2
13. Description of dropout rate	2	2	2	2
14. Similarity of groups at baseline for important prognostic indicators	1	0	1	1
15. Role of cointerventions	1	1	1	1
V. Randomization				
16. Method of randomization	2	2	2	2
VI. Allocation concealment				
17. Concealed treatment allocation	2	2	2	2
VII. Blinding				
18. Patient blinding	1	1	1	1
19. Care provider blinding	1	1	1	1
20. Outcome assessor blinding	0	0	0	0
VIII. Conflicts of interest				
21. Funding and sponsorship	2	2	2	2
22. Conflicts of interest	3	3	3	3
Score	42	43	44	42

**Table 4:** Characteristics of included studies on cervical epidural injections in neck pain.

Study/study type Methodologic quality scoring	Participants and interventions	Outcome measure	Follow-up period	Conclusions
Manchikanti et al. (36)/RCT Quality scores: Cochrane = 11/13; IPM − QRB = 42/48	*N* = 60 Epidural injections without steroid, *n* = 30 Epidural injections with steroid, *n* = 30 Group I: cervical interlaminar epidural injections with 5 ml of 0.5% lidocaine Group II: cervical interlaminar epidural injections with 4 ml of 0.5% lidocaine mixed with 1 mL or 6 mg of nonparticulate betamethasone	NDI, NRS, opioid intake, work status	1 year	Cervical interlaminar epidural injections had an efficacy of 71.5% for pain reduction and improvement in functional status for neck pain.

Manchikanti et al. (37)/RCT Quality scores: Cochrane = 12/13; IPM − QRB = 43/48	*N* = 120 Epidural injections without steroid, *n* = 60 Epidural injections with steroid, *n* = 60 Group I: cervical interlaminar epidural injections with 5 ml of 0.5% lidocaine Group II: cervical interlaminar epidural injections with 4 ml of 0.5% lidocaine mixed with 1 ml or 6 mg of nonparticulate betamethasone	NDI, NRS, opioid intake, work status	2 years	Cervical interlaminar epidural injections for chronic neck pain was effective in 72% of patients in group I and 68% of patients in group 2.

Manchikanti et al. (38)/RCT Quality scores: Cochrane = 11/13 IPM − QRB = 44/48	*N* = 120 Epidural injections without steroid, *n* = 60 Epidural injections with steroid, *n* = 60 Group I: cervical interlaminar epidural injections with 5 mL of 0.5% lidocaine Group II: cervical interlaminar epidural injections with 4 ml of 0.5% lidocaine mixed with 1 ml or 6 mg of nonparticulate betamethasone	NDI, NRS, opioid intake, work status	2 years	Cervical epidural injections of local anesthetic with or without steroids were effective in 71% of patients.

Manchikanti et al. (39)/RCT Quality scores: Cochrane = 11/13 IPM − QRB = 42/48	*N* = 116 Epidural injections without steroid, *n* = 58 Epidural injections with steroid, *n* = 58 Group I: cervical interlaminar epidural injections with 5 ml of 0.5% lidocaine Group II: cervical interlaminar epidural injections with 4 ml of 0.5% lidocaine mixed with 6 mg of nonparticulate betamethasone	NDI, NRS, work status	2 years	Cervical interlaminar epidural injections for chronic neck pain alleviated pain relief and improved functional status by ≥50% in 69% of patients in group I and 71% of patients in group 2 at the 2-year follow-up.

IPM-QRB: Interventional Pain Management Techniques-Quality Appraisal of Reliability and Risk of Bias Assessment; NDI: Neck Disability Index; NRS: numeric rating scale; RCT: randomized controlled trial.
